# Comparative Study and Simulation of Capacitive Sensors in Microfluidic Channels for Sensitive Red Blood Cell Detection

**DOI:** 10.3390/mi13101654

**Published:** 2022-09-30

**Authors:** Wei Hu, Bingxing Wu, Soumya K. Srivastava, Suat Utku Ay

**Affiliations:** 1 Thermo Fisher Scientific, Jinke Road 2537, Pudong District, Shanghai 201206, China; 2 Department of Chemical and Biomedical Engineering, West Virginia University, Morgantown, WV 26506-6102, USA; 3 Department of Electrical and Computer Engineering, University of Idaho, Moscow, ID 83844-1023, USA

**Keywords:** microfluidics, lab-on-chip, capacitive sensor, interdigital capacitor sensor (IDC)

## Abstract

Microfluidics provides an indispensable platform for combining analytical operations such as sample preparation, mixing, separation/enrichment, and detection onto a single compact platform, defined as a lab-on-a-chip (LOC) device with applicability in biomedical and life science applications. Due to its ease of integration, 1D interdigital capacitive (IDC) sensors have been used in microfluidic platforms to detect particles of interest. This paper presents a comparative study on the use of capacitive sensors for microfluidic devices to detect bioparticles, more specifically red blood cells (RBCs). The detection sensitivities of 1D, 2D, and 3D capacitive sensors were determined by simulation using COMSOL Multiphysics^®^ v5.5. A water-filled 25 μm × 25 μm PDMS microfluidic channel was used with different sizes (5–10 μm) of red blood cells passing across the capacitive sensor regions. The conformal mapping was used for translating the 1D IDC sensor dimensions into equivalent 2D/3D parallel plate capacitance (PPC) sensor dimensions, creating similar absolute sensor capacitance. The detection sensitivity of each capacitive sensor is determined, and a new 3D PPC sensor structure was proposed to improve the sensitivity for high-resolution RBC detection in microfluidic channels. Proposed 2D and 3D sensors provide a 3× to 20× improvement in sensitivity compared to the standard 1D IDC structures, achieving a 100 aF capacitance difference when a healthy RBC passes in the structure.

## 1. Introduction

**Figure 1:**
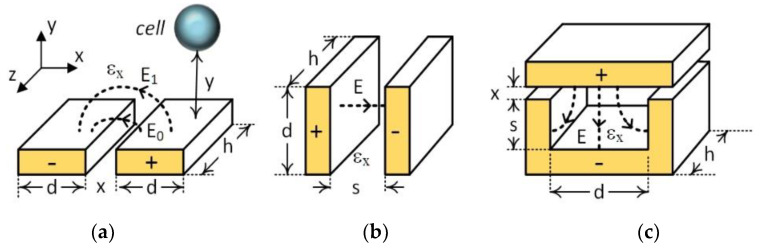
Three Capacitive sensor structures and the electric field distributions in; (**a**) 1D coplanar interdigital capacitor (IDC), (**b**) 2D parallel plate capacitor (PPC), (**c**) 3D PPC.

The use of capacitive structures as sensors has been widely investigated and published since 1960s, with them finding applications in many fields [1,2,3,4,5], recently including biomedical and life science areas [6,7,8,9]. Among them, coplanar interdigital capacitor (IDC) can be considered as a one-dimensional (1D) sensing structure that utilizes fringing electric fields on interdigitated electrodes providing a one-sided investigation of the materials and any particulates passing above them, as shown in Figure 1a. These coplanar IDCs are the 1D mapping of the 2D parallel plate capacitors (PPCs) through conformal mapping [10,11,12]. Two- and three-dimensional parallel plate capacitors, as shown in Figure 1b,c, date back to the early days of electricity and electromagnetics [13,14]. They are used as sensors either by detecting changes in the suspending medium properties (i.e., permittivity, ε_x_) in where the electric field (E) is formed or by detecting changes that occurred in the physical properties of the capacitor structure (i.e., separation of the plates, s). For example, micro-machined accelerometers use suspended capacitor plates that can be displaced proportionally to the acceleration rate modulating plate separation (s) and hence the capacitance of the sensor [15,16]. In the case of microfluidic devices, the permittivity of the suspending medium in the microfluidic channel where the capacitive sensor is placed changes either due to the changes in the environmental conditions where the device is deployed (i.e., pressure, temperature, humidity) or by particles with different permittivity passing in the region of electric fields formed between the sensor plates. In all cases, the accurate measurement of the sensor capacitance will detect and quantify the main phenomena causing the change.

One-dimensional IDCs are widely used in the microfluidic platform due to their low-cost, low-drift, stable characteristics, easy fabrication and integration, and label-free detection capabilities [17,18,19]. Moreover, they have the lowest complexity among all the other bio-detection sensors, enabling compact point-of-care lab-on-chip (LoC) devices [20]. The main disadvantage of 1D IDCs compared with 2D and 3D PPCs for cell detection applications in microfluidic channels is their low sensitivity due to their short detection range in the inspected suspending medium. The detection range (y in Figure 1a) depends on the strength of the electric field in the Y-direction in the medium, which is controlled by the applied potential difference between the IDC electrodes. The higher the difference is, the higher the extension of the electric fields in the y-direction will be, yielding better sensitivity for IDCs. However, higher potential differences increase electric field strength close to the surface of the IDCs in microfluidic channels (E_0_ > E_1_), which inadvertently damages cell membranes and causes lysing [21]. Thus, trade-offs exist among frequency and magnitude of applied potentials and detection sensitivity in 1D IDC sensors. As opposed to 1D IDCs, 2D and 3D PPCs structures provide lower operation potential and better sensitivity, as presented in this paper. The main reasons why 2D and 3D sensor structures are not preferred in today’s microfluidic platforms are the manufacturability and integration issues of these type of sensors have, therefore making LoC devices expensive.

## 2. Detection Principle

One sensing principle of molecular detectors is that they measure the induction of charge as the by-product of the binding process between the receptor and the analyte under investigation [18]. Moreover, without using a receptor, a simple capacitive sensor with only electrodes can also be used to detect particles. Ciccarella et al. [22] showed a capacitive airborne particle detector in which the particles on the IDC electrodes cause the change in the dielectric constant of the volume surrounded by the air. As a result, the capacitance of the structure changes, and it can be used to quantify the size of the particles. This paper builds sensors for detecting bioparticles (red blood cells (RBCs)) in microfluidic channels based on a similar idea.

In COMSOL Multiphysics^®^ software v5.5, the capacitance can be calculated using Equation (1) in the electrostatic simulation module [23]. The capacitance is determined by the charge accumulated on the electrodes where a fixed voltage difference (ΔV = 1 V) is applied between them. Moreover, the accumulated charge (Q) is proportional to the dielectric constant of the material (ε_r_) between the electrodes. When RBCs (ε_r_ ≈ 46.9) pass through the sensor electrodes, the medium of water (ε_r_ = 80) surrounding the electrodes will be altered. Thus, it yields a decrease in capacitance as shown in Equation (2), where Q_particle_ and Q_init_ are the amounts of charge accumulated on the sensor electrodes while RBCs are passing through the microfluidic sensor region and before the RBCs enter the channel (i.e., initial condition with only the presence of water medium), respectively.

(1) $$C=\frac{Q}{\Delta V}$$

(2) $$\mathrm{dC}=\frac{\mathrm{dQ}}{\Delta V}=\frac{Q_{\mathrm{particle}}-Q_{\mathrm{init}}}{\Delta V}$$

The initial capacitance value for 1D coplanar IDC and 2D PPC are set close to each other by tuning the parameters of the electrodes (i.e., the height and width of the electrodes). To find approximated parameters that yield similar sensor capacitance in both structures, the conformal mapping technique is used to map the electric field of IDC to PPC to create an equivalent PPC model, which makes it feasible to find the capacitance of an IDC. The results from the simulations were utilized to design novel 3D structures (L and Ω) for achieving improved sensitivity to RBCs in different locations of the sensing areas within the microfluidic channel.

## 3. Conformal Mapping

**Figure 2:**
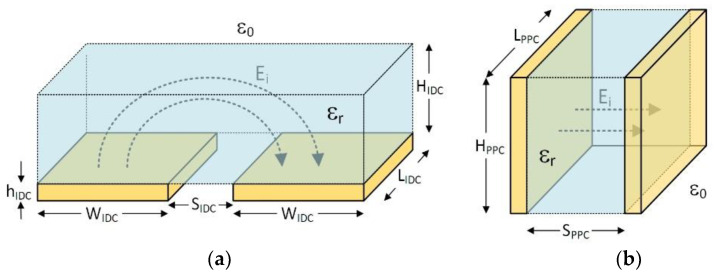
The dimensions used to map an (**a**) IDC structure to a (**b**) PPC structure.

To calculate the capacitance of PPC, it is customary to use conformal mapping. The use of conformal mapping based on the Christoffel–Schwarz transformation [24] makes it possible to transform the IDC structures shown in Figure 2a to PPC structure in Figure 2b. The following equations [25,26] determines each structure’s dimensions, excluding capacitor fringing fields and assuming thickness of the ICD plates (h_IDC_) is significantly smaller than other dimensions.

(3) $$C_{\mathrm{IDC}}=\frac{\varepsilon_{0}\varepsilon_{r}}{2}\cdot \frac{K\left(\;k^{\prime }\right)}{K\left(k\right)}\cdot L_{\mathrm{IDC}}$$

(4) $$C_{\mathrm{PPC}}=\frac{\varepsilon_{0}\varepsilon_{r}\cdot H_{\mathrm{PPC}}}{S_{\mathrm{PPC}}}\cdot L_{\mathrm{PPC}}$$

(5) $$\frac{H_{\mathrm{PPC}}}{S_{\mathrm{PPC}}}=\frac{K\left(\;k^{\prime }\right)}{K\left(k\right)}=\left\{\begin{array}{c} \pi^{-1}\cdot \ln\left(2\cdot \frac{1+\sqrt{\;k^{\prime }}}{1-\sqrt{\;k^{\prime }}}\right){\;\mathrm{for}\;k}^{2}\leq 0.5\\ \pi \cdot \ln\left(2\cdot \frac{1+\sqrt{k}}{1-\sqrt{k}}\right){\;\mathrm{for}\;k}^{2}\geq 0.5 \end{array}\right.$$

where K(k) is the elliptic integral of the first kind, and k and k’ can be written as:

(6) $$k=\sqrt{1-\frac{{\left(\frac{S_{\mathrm{IDC}}}{2}\right)}^{2}}{{\left(\frac{S_{\mathrm{IDC}}}{2}+W_{\mathrm{IDC}}\right)}^{2}}}$$

(7) $$\;k^{\prime }=\sqrt{1-k^{2}}$$

Here, ε_0_ is the permittivity of free space, and ε_r_ is the dielectric constant of the medium in which the electric field is confined.

This simple model ignores the fringing fields of the capacitors. Additionally, it assumes the capacitor plates are suspended in the air. However, capacitive sensors in microfluidic devices are sandwiched in supporting material [27] (i.e., glass, PDMS, etc.), forming multi-layer structures with multi-finger IDCs and finite plate thicknesses. Several models have been proposed for accurate calculation of the capacitance of the IDC sensors over the years [28,29,30]. For initial design calculations, additional capacitances caused by the multi-layer dielectrics, fringing fields, and plate thicknesses were ignored as both IDC and PPC structures are built-in same microfluidic channels formed in PDMS material and filled with water (ε_r_ = 80) as suspending medium that has much higher dielectric constant than that of the other two media, the PDMS substrate, and air.

## 4. Capacitive Sensor Design

Different sizes of capacitive sensors were designed considering Polydimethylsiloxane (PDMS) polymer for microfluidic channels to evaluate the performance of 1D IDC and 2D/3D PPC sensor structures. It was assumed that the width and height of the channel were 25 μm. A design and mapping process was developed to calculate the initial capacitance and dimensions of the three structures.

**Figure 3:**
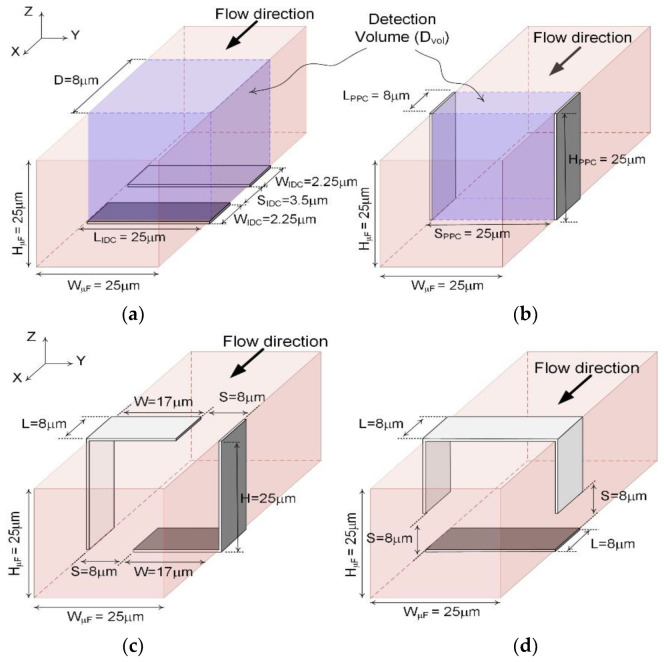
Design parameters of capacitive sensor structures in 25 μm microfluidic channel; (**a**) 1D coplanar IDC, (**b**) 2D PPC, (**c**) L-shape 3D PPC, (**d**) Ω-shape 3D PPC.

Equations (3)–(7) are used to calculate the approximated dimensions of coplanar 1D IDC and 2D/3D PPCs for a 25 μm channel through the following procedure:

1. For 2D/3D PPC structures, H_PPC_ = S_PPC_ is fixed by the channel height (H_μF_) and width (W_μF_) to 25 μm, while the IDC length is set to the channel width (L_IDC_ = W_μF_). Thus, using Equations (3), (4) and (8) is obtained:
  (8) $$\frac{1}{2}\cdot \frac{K\left(\;k^{\prime }\right)}{K\left(k\right)}\cdot W_{\mu F}=L_{\mathrm{PPC}}$$
  Using Equations (5)–(7), S_IDC_ and W_IDC_ can be written in terms of given L_PPC_.
2. Assuming capacitive sensors occupy the same detection volume (D_vol_) in microfluidic channel, we can use;
  (9) $$S_{\mathrm{PPC}}=H_{\mathrm{PPC}}=H_{\mathrm{IDC}}=L_{\mathrm{IDC}}=W_{\mu F}=H_{\mu F}$$
  (10) $$2\times W_{\mathrm{IDC}}+S_{\mathrm{IDC}}=L_{\mathrm{PPC}}$$
3. Length of the PPC structure is set to the maximum size of the bioparticle. Thus, L_PPC_ is set to 8 μm since healthy RBCs average diameter is typically between 7.5 μm and 8.7 μm.
4. Using Equations (5)–(8) and (10), dimensions of the IDC can be determined, resulting in similar capacitance values, as shown in Figure 3a,b for a 25 μm microfluidic channel.

Maintaining detection volume (D_vol_), capacitor plates of the 2D PPC sensor were extended to form the two 3D PPC structures (L-shape and Ω-shape), as shown in Figure 3c,d. COMSOL Multiphysics^®^ was used to tune the dimensions to determine the initial capacitances of each design for both channel sizes. In 25 μm design, W_IDC_ and S_IDC_ are designed to be 2.25 μm and 3.5 μm so that IDC has the same sensing area (D = 8 μm) and volume with a similar initial capacitance value as the 2D/3D PPC sensors. The initial capacitances (C_init_) of the 1D and 2D/3D designs are listed in Table 1. These structures were setup up in COMSOL with an *extra fine* mesh size and the electrostatic module was used to operate the simulation.

## 5. Red Blood Cell (RBC) Properties for Simulation

The amplitude and frequency of the electric field inside the capacitive sensors play a critical role in effectively detecting bioparticles and RBCs [31]. This is also necessary for accurate simulation of their effect on the capacitance of these sensors when they are deployed in microfluidic channels. It was found that the RBC behaves as an insulator at very low frequencies (<1 kHz), blocking the electric field, mainly revealing the property of the RBC membrane. While at high frequency (>1 MHz), the RBC membrane behaves as a short circuit which mainly reveals only the conductive material of the intercellular medium [32]. The method to select the frequency range for cell detection can be traced back to the Foster and Schwan [33] cell model, which describes the equivalent circuits for cell membrane undercharging. In terms of the cell-to-sensor interaction, cells are attracted to the sensor areas [34] due to the Maxwell—Wagner polarization, which would occur between 600 kHz and 1.1 MHz for RBCs suspended in serum (generalized as water) (ε_r_ = 80) within the microfluidic channels [35]. Thus, it is desirable to operate capacitive sensors below this frequency range (i.e., between 10 kHz and 100 kHz as suggested for RBC detection [36]), in which the cell can be considered as an insulator while providing good sensitivity and preventing immobilization or trapping of the cells on sensor plates [35]. In our simulation, we chose DC frequency to examine the cells by evaluating the change in capacitance.

The amplitude of the applied electric field should be less than the critical electric field strength of 2.1 kV/cm to prevent possible damage to the cell membrane [37]. Hence, to improve the sensitivity and not trap the cells in the channel, the bias voltage of 1 V was chosen for simulation, which resulted in <1.5 kV/cm electric field strength for a 25 μm microfluidic channel.

Lastly, the dielectric properties of RBC can also be obtained through the shell model [38] with the permittivity and conductivity of cell’s membrane and cytoplasm in [36]. As a result, in COMSOL simulation, the RBC cell’s relative permittivity and conductivity were calculated as ε_r_ = 46.98 and σ = 0.7 S/m, respectively. The properties of the rest materials are listed in Table 2.

## 6. Simulation Results

The size and location of the bioparticle (RBC in this case) placed in and around the detection volume (D_vol_) of the microfluidic channel were varied using the coordinate system as shown in Figure 3. The capacitance of the sensors was determined with and without bioparticles in the detection volume by building the structures in the COMSOL Multiphysics^®^ and by performing the electrostatic simulation using Equation (1). Besides the absolute capacitance variation (dC) of sensors, the relative sensitivity (S_R_) and size detection sensitivity (S_S_) of each sensor were also determined using the following equations:

(11) $$S_{R}=\mathrm{dC}/C_{\mathrm{init}}\left(\mathrm{in}\;\%\right)$$

(12) $$S_{S}=\mathrm{dC}/\mathrm{dr}\;\left(\mathrm{in}\;\mathrm{aF}/\mu m\right)$$

where C_init_ is the initial capacitance of the sensor without bioparticles in the channel, dr is the size change, and dC is the change in capacitance value when the bioparticle is in or close to the detection volume.

Absolute change of the capacitance value (dC) of the sensor is important to assess the capabilities of the structures. This is because the initial capacitance value of the sensor and the long wiring capacitances of the microfluidic system can easily be measured and/or canceled through readout techniques such as DC correlated double sampling [39] or AC chopping techniques [40]. Thus, more significant absolute changes result in better resolution in RBCs detection in microfluidic channels. In the simulation, the size of a single RBC and its location to evaluate the performance of different structures were varied using the Equations (11) and (12).

A. *Different RBC Size*

**Figure 4:**
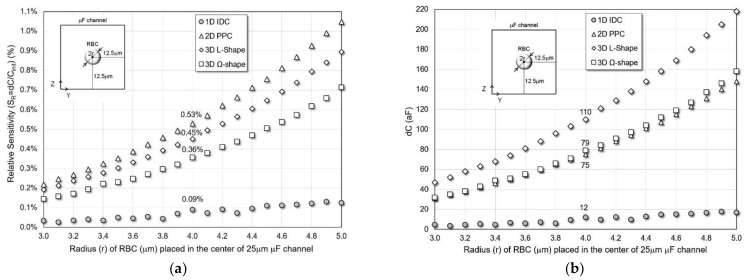
(**a**) Relative sensitivity and (**b**) capacitance change of the sensors for varying size of RBC cells placed in the center of a 25 μm microfluidic channel.

The radius of the bioparticle, the RBC, is varied from 3 μm to 5 μm and placed in the center of the 25 μm channel. As observed in Figure 4a, the sensitivity increases with the radius. Two-dimensional PPC and 3D L-shaped sensors show the best absolute sensitivity of different size RBCs. Meanwhile, 3D L-shaped structure provides the largest size sensitivity which is critical for detection resolution. For a normal size RBC (r = 4 μm), 2D PPC and L-shaped 3D sensors have 0.53% and 0.45% relative sensitivity in 25 μm channels. In all cases, 2D and 3D structures have higher sensitivity than the standard 1D coplanar IDC sensor.

Figure 4b shows the simulated dC of the structures in the 25 μm channel. Comparing the dC among the four structures, we found that the 3D L-shape structure has the best dC value of 220 aF for a normal size RBC in the 25 μm channel, achieving a size sensitivity of 85.5 aF/μm which is calculated by averaging S_s_ values for r between 3 and 5 μm. Two-dimensional PPC and 3D Ω-shaped structures have similar 64 aF/μm size sensitivity and dC of 75 aF and 79 aF, respectively. While the 1D IDC structure provides significantly smaller dC change requiring zF detection capabilities [22,41].

B. *Different Z-Locations*

**Figure 5:**
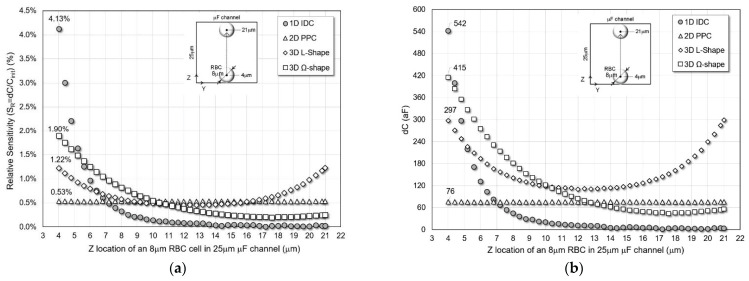
(**a**) Relative sensitivity and (**b**) capacitance change of the sensors for varying Z location of 8 μm RBC cell in 25 μm microfluidic channel.

The location of a typical size RBC (r = 4 μm) is changed in the Z direction from bottom to top without contacting the sensors plates, while the other two coordinates, X and Y, are fixed at the center of the channel. The four sensors’ simulated sensitivity and absolute capacitance change are shown in Figure 5 for the 25 μm microfluidic channel. 

The 1D IDC has the highest peak sensitivity and dC of 4.13% and 542 aF in a 25 μm channel when the RBC is close to the bottom of the channel or just above the IDC electrodes. As the distance between the RBC and the IDC electrodes placed on the XY-plane increases, the sensitivity and dC sharply decreases compared to the other designs. Thus, 1D IDC achieves the worst average relative sensitivity and dC of 0.41% and 55 aF. The 2D PPC possesses the second least sensitivity and dC with constant values of 0.53% and 76 aF mainly because the 2D PPC electrodes are placed on XZ-plane.

The L-shape structure maintains higher average sensitivity and dC values of 0.67% and 163 aF with peak values of 1.22% and 297 aF, respectively. This is mainly because of the electrode spacing at the top and bottom corners of the channel creating a more uniform and symmetric electric field. The 3D Ω-shaped structure has the second-best performance surpassing L-shaped sensor characteristics close to the bottom of the channel with average sensitivity and dC values of 0.58% and 127 aF and peak values of 1.9% and 415 aF. This is because the electrode spacing only occurs close to the bottom of the channels.

C. *Different Y-Locations*

**Figure 6:**
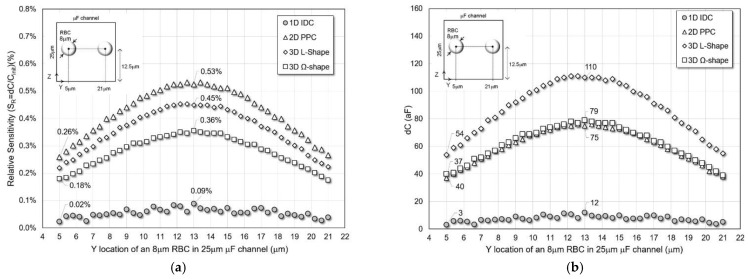
(**a**) Relative sensitivity and (**b**) capacitance change of the sensors for varying Y-location of an 8 μm RBC cell in a 25 μm microfluidic channel.

The location of a typical size RBC (r = 4 μm) is changed in Y-direction from left to right without contacting the sensors plates, while the other two coordinates, X and Z, are fixed at the center of the channel. The four sensors’ simulated relative sensitivity and absolute capacitance change are shown in Figure 6 for a 25 μm microfluidic channel.

All 2D and 3D structures have electrodes on the YZ-plane sidewalls of the microfluidic channel and have similar performance trends for sensitivity and dC. The 3D L-shaped sensor has an average sensitivity and dC of 0.36% and 88 aF with peak values of 0.45% and 110 aF, respectively. Two-dimensional PPC has a higher average and peak relative sensitivity of 0.42% and 0.53%, respectively. However, the average and peak dC are lower than the 3D L-shaped sensor with 60 aF and 75 aF, respectively. Three-dimensional Ω-shape sensors are inferior to 2D PPD and 3D L-shape sensors, with average relative sensitivity and dC of 0.28% and 62 aF and peak values of 0.36% and 79 aF, respectively. It is much better than the 1D IDC structure, with average and peak performance values of 0.056% and 0.09% sensitivity and 7.6 aF and 12 aF of dC.

D. *Different X-Locations*

**Figure 7:**
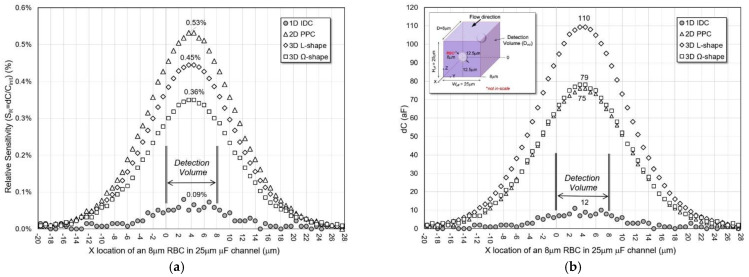
(**a**) Relative sensitivity and (**b**) capacitance change of the sensors for varying X-location of an 8 μm RBC cell in a 25 μm microfluidic channel.

A typical size RBC (r = 4 μm) traveling in X-direction in the middle of a 25 μm microfluidic channel is simulated, as shown in Figure 7. As fringing fields play a critical role in the capacitance of 2D and 3D sensors, RBC is moved from −20 μm to +20 μm of the detection volume in the X-direction to quantify dC and S_R_.

Simulations confirmed that 2D/3D sensors are more effective than the 1D IDC sensors in and beyond their detection volumes. Among them, 2D PPC shows higher sensitivity. However, the 3D L-shape sensor provides the largest average (within detection volume) and peak dC values of 99 aF and 110 aF, respectively. Additionally, among the four structures, it has the largest ratio of dC between the RBC in and beyond the detection volume. Through this simulation, detecting particles are possible beyond the sensor sites’ detection volume for 2D/3D structures.

E. *E-Field Distributions*

**Figure 8:**
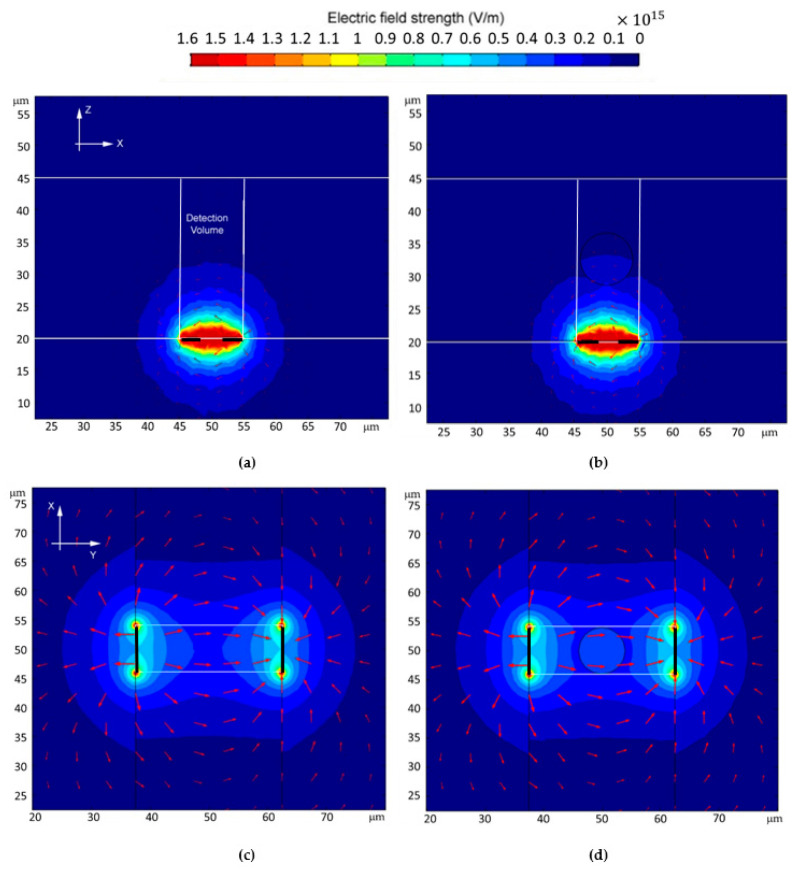
Electric field strength in the detection volume with and without 8 μm RBC in 25 μm microfluidic channel for; (**a**,**b**) 1D IDC (**c**,**d**) 2D PPC.

**Figure 9:**
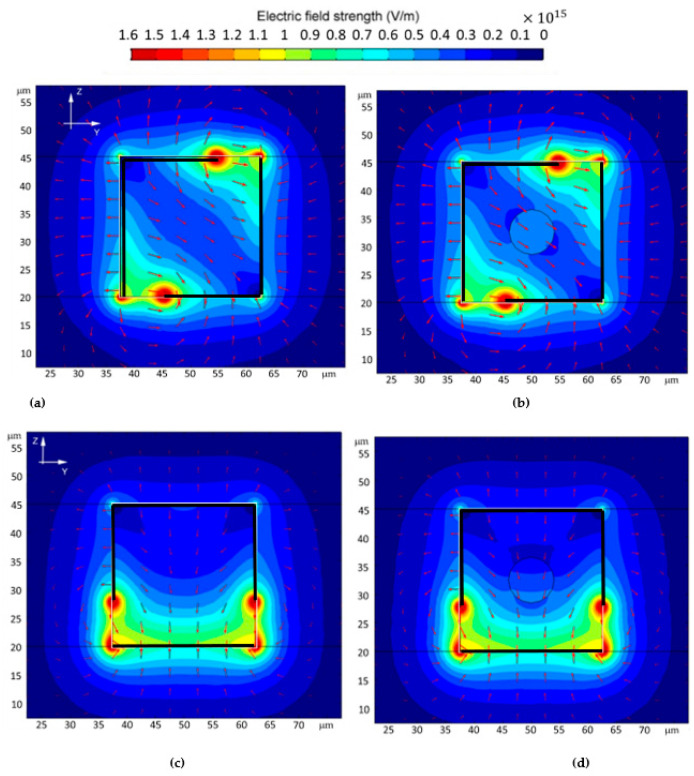
Electric field strength in the detection volume with and without 8 μm RBC in 25 μm microfluidic channel for; (**a**,**b**) 3D L-shaped (**c**,**d**) 3D Ω-shaped.

Electric field distribution inside the detection volume is a good indication of whether a sensor is sensitive to the bioparticles located inside the channel. The electric field strength in V/m for the four sensor structures with and without RBCs are shown in Figure 8 and Figure 9. For the same applied bias voltage of 1 V, E-field reaches and coverage of 2D/3D sensors are inclusive, which explains why they are more sensitive than the 1D IDC structure. One-dimensional IDC also has a high concentration of the E-field close to the electrodes, possibly causing undesirable field distribution on nearby RBS and, in cases of high voltage, breaking down the cell barrier, causing lysing [21].

## 7. Conclusions

A comparative study of the use of 1D, 2D, and 3D capacitive sensors in microfluidic devices to detect bioparticles was performed. A procedure to design and map a 1D IDC structure to 2D and 3D capacitive structures was developed. Proposed designs were simulated using COMSOL Multiphysics^®^ v5.5. The simulations indicate that the proposed 3D structures have larger dC values in most scenarios, and among the 3D structures, the L-shape sensor is a possible candidate for the sensor design due to it having the largest dC as the RBC approaches the center of the channel. Proposed 2D and 3D sensors provide a 3× to 20× improvement in sensitivity compared to the standard 1D IDC structures, achieving a 100 aF capacitance difference when a healthy RBC passes in the structure.

The symmetric electric field in L-shaped structure contributes to a wide and uniform sensing capability. Furthermore, if combining the sensor design with flow control techniques such as hydrodynamic focusing [42], it can identify the capacitance differences due to size or dielectric property of individual cells in a stream. A fast cell counting application could also be possible due to the extent of the sensing at the center of the channel by properly sampling the dC and developing intelligent algorithms. However, the simulation results presented in this paper were obtained statically and hence did not consider the influence of the real device conditions such as flux and dielectrophoretic effects [43]. For future work, a dynamic simulation is important to evaluate the capacitance change as RBC flows through a flux in the channel.

## Figures and Tables

**Table 1:** Initial capacitances (C_init_) of the 1D and 2D/3D designs.

Definition	1D IDC	2D PPC	3D L-Shape	3D Ω-Shape
Initial Capacitance C_init_ (fF)	13.7	14.3	24.6	22.3

**Table 2:** Dielectric constants and conductivity of materials used for simulations.

Definition	Material	Dielectric Constant	Conductivity (S/m)
Microfluidic Base	PDMS	2.7	insulator
Blood Serum	Water	80	5.5 × 10^−6^
Sensor Plates	Platinum	1.0	8.9 × 10^+6^
Detected Biomaterial	RBC	46.98	0.70

## Data Availability

Not applicable.
